# Relationship between Violent Behavior and Repeated Weight-Loss Dieting among Female Adolescents in Japan

**DOI:** 10.1371/journal.pone.0107744

**Published:** 2014-09-11

**Authors:** Nao Shiraishi, Atsushi Nishida, Shinji Shimodera, Tsukasa Sasaki, Norihito Oshima, Norio Watanabe, Tatsuo Akechi, Toshiaki A. Furukawa, Yuji Okazaki

**Affiliations:** 1 Department of Psychiatry and Cognitive-Behavioral Medicine, Nagoya City University Graduate School of Medical Sciences, Mizuho-cho, Mizuho-ku, Nagoya, Japan; 2 Department of Psychiatry and Behavioral Science, Tokyo Metropolitan Institute of Medical Science, Setagaya-ku, Tokyo, Japan; 3 Department of Neuropsychiatry, Kochi Medical School, Kochi University, Nanngoku, Kohchi, Japan; 4 Laboratory of Health Education, Graduate School of Education, the University of Tokyo, Bunkyo, Tokyo, Japan; 5 Office for Mental Health Support, Division for Counseling and Support, The University of Tokyo, Bunkyo, Tokyo, Japan; 6 Department of Clinical Epidemiology, Translational Medical Center, National Center of Neurology & Psychiatry, Kodaira, Tokyo, Japan; 7 Department of Health Promotion and Human Behavior, Kyoto University Graduate School of Medicine/School of Public Health, Yoshida Konoe-cho, Sakyo-ku, Kyoto, Japan; 8 Tokyo Metropolitan Matsuzawa Hospital, Setagaya, Tokyo, Japan; The University of Queensland, Australia

## Abstract

**Purpose:**

To examine whether interpersonal violence perpetration and violence toward objects are associated with body mass index (BMI), body weight perception (BWP), and repeated weight-loss dieting in female adolescents.

**Methods:**

A cross-sectional survey using a self-report questionnaire was performed evaluating interpersonal violence perpetration, violence toward objects, the number of diets, BMI, BWP, the 12-item General Health Questionnaire (GHQ-12), victimization, substance use, and other psychosocial variables among 9,112 Japanese females aged between 12–18 years. Logistic regression analysis was conducted to analyze the contribution of BMI, BWP, and weight-control behavior to the incidence of violent behavior, while controlling for potential confounding factors.

**Results:**

The number of diets was associated with both interpersonal violence perpetration (OR = 1.18, 95% CI 1.08–1.29, *p*<0.001) and violence toward objects (OR = 1.34, 95% CI 1.24–1.45, *p*<0.001), after adjusting for age, BMI, BWP, the GHQ-12 total score, victimization, and substance use. In terms of BMI and BWP, the “overweight” BWP was associated with violence toward objects (OR = 1.29, 95% CI 1.07–1.54, *p*<0.05). On the other hand, the “Underweight” and “Slightly underweight” BMI were related to violence toward objects [(OR = 1.28, 95% CI 1.01–1.62, *p*<0.05) and (OR = 1.27, 95% CI 1.07–1.51, *p*<0.05), respectively]. The “Underweight” BWP was related to interpersonal violence perpetration (OR = 2.30, 95% CI 1.38–3.84, *p*<0.05).

**Conclusions:**

The cumulative number of diets is associated with violent behavior in female adolescents. In addition, underweight BMI and extreme BWP are associated with violent behavior.

## Introduction

Violence among young people is a major social issue around the world [1], [2]. In the United Sates, 32% of high school students are involved in a physical fight and no fewer than 19% of female students physically abused someone at least once in the previous year [3]. The annual statistical survey for problematic behaviors of Japanese students shows that the incidence of violent behavior, such as interpersonal violence perpetration and property destruction, has risen approximately 1.5 times in the past decade [4]. Physical violence is likely to impair the quality of life of adolescents in family environment, peer relationships, and school perceptions [5]. The risk factors behind violence may include low academic performance [6], [7], parent–child and peer relationship [8]–[10], victimization [7], [10], illegal drug use [6], [7], [9], [10], smoking [11], illegal alcohol consumption [6], [8], [10], and adolescent psychiatric disorders such as depression [8], attention-deficit/hyperactivity disorder (ADHD) and conduct disorder [9], [12].

Weight-loss dieting is a frequent behavior among adolescents in industrialized countries, regardless of their race or nationality [2], [3]. The prevalence of dieting is positively associated with a higher BMI [13]. More females than males have frequent experiences of dieting, across junior and senior high school students of all ages [2], [13], [14]. The reason for the difference between genders is that female adolescents commonly have much greater body-image dissatisfaction than their male counterparts [14]–[16]. Moreover, 13% of female dieters engage in maladaptive weight-loss behaviors (self-induced vomiting and binge eating) in the United States [13]. Exposure to violence, especially during childhood [17], [18], and several adolescent psychiatric disorders (e.g., depression [13], [19] or ADHD [20], [21]) also influence BMI, BWP or weight-control behavior negatively.

Chronic dieting can cause negative effects on physical and mental health [22]. It can result in delayed growth [23], electrolyte imbalance, cardiac dysfunction [24], and morbidity due to nutritional deficiencies such as iron deficiency anemia [22], [25]. Furthermore, chronic dieting can affect mental health because it is related to persistent irritability [26], emotional dysregulation, poor impulse control [27], [28], low self-esteem [24], depression, and anxiety [22], [29], [30]. Similarly, physical violence is associated with increased irritability and a bad temperament [31]. These findings led us to hypothesize that frustration resulting from repeated dieting is associated with an increased probability of violence. It is known that perceived weight status and disordered eating including self-induced vomiting are related to violent behavior [32]. However, it has not yet been reported whether there is an association between violent behavior and repeated dieting. Thus, we aimed to examine the contribution of BMI, BWP, and weight-control behavior to the incidence of violent behavior in adolescents. Since the distribution of the number of diets was different between the sexes, the present study was performed only among females. Our hypothesis is that interpersonal violence perpetration and violence toward objects are associated with BMI, BWP, and repeated weight-loss dieting in female adolescents.

## Methods

### Ethics statement

The study was approved by the ethics committees of the Tokyo Institute of Psychiatry, the Kochi Medical School, and the Mie University School of Medicine and was conducted in accordance with the principles of the Helsinki Declaration. We complied with Japan's Ethical Guidelines for Epidemiological Research.

### Subjects and procedures

We conducted a cross-sectional survey between 2008 and 2009 that investigated the psychopathology in adolescence. A detailed description of our sampling method has previously been reported and is only briefly discussed here [33]. For the survey, students were recruited from 45 public junior high schools (7^th^–9^th^ grade) and 28 public high schools (10^th^–12^th^ grade) in Kochi Prefecture and Tsu City, Japan. The populations of Kochi Prefecture and Tsu City are approximately 750,000 and 290,000, respectively; both of these areas are located in the mid-west part of Japan. Kochi Prefecture has urban and rural regions that surround a centrally located prefectural capital. Tsu City, which is the prefectural capital of Mie Prefecture, is a typical medium-size city in Japan. Therefore, these two areas are representative for general adolescent population. According to the Japanese law, junior high school is a part of the compulsory education system, whereas high school is not.

Our survey followed a standard procedure. School principals were approached about participating in the study. The principals discussed participation with teachers and parents. For schools that agreed to participate, teachers distributed questionnaires and envelopes to their students. At the time of distribution, the teachers explained to the students (1) that participation in the study was anonymous and voluntary, and (2) that strict confidentiality would be maintained. The teachers also explained that the completed questionnaire should be sealed in the provided envelope. Finally, survey staff members went to each school and collected the sealed questionnaires.

### Measures

The self-report questionnaire included the following items: (1) cognitive and behavioral problems including interpersonal violence perpetration, violence toward objects, number of diets, body weight perception (BWP), and self-induced vomiting for the purpose of dieting; (2) the Japanese version of the 12-item General Health Questionnaire (GHQ-12); and (3) other variables including demographical characteristics, height, and weight.

#### Interpersonal violence perpetration and violence toward objects

Interpersonal violence perpetration and violence toward objects that occurred in the previous year were assessed using two questions: “Have you physically abused someone in your family or your friends within the past year?” and “Have you been extremely frustrated and damaged something within the past year?” Each student had a choice of two answers: “yes” or “no.” Self-harm was not included in violent behavior in this study.

#### Number of diets

Dieting experience was assessed with the question: “Have you ever gone on a weight-loss diet?” Students could select “yes” or “no.” “No” was defined as “never,” and “yes” indicated the number of diets: “How many times have you ever gone on a weight-loss diet?” The response to this question was then categorized into one of the following groups: “1–3 times,” “4–7 times,” “8–12 times,” and “more than 13 times.”

#### Body Mass Index

BMI was calculated from a student's weight (kg) divided by their height (m^2^), which were based on a self-reported height and weight. Evidence shows that self-reported BMIs are highly correlated with their measured counterparts in female adolescents aged 12–18 years (*r* = 0.85) [34]. Investigators (NS and NW) determined the exclusion of unlikely height and weight from the analyses by reference to the annual school health statistical surveys of the Ministry of Education, Culture, Sports, Science, and Technology in Japan [35].

The BMI was classified into five categories: “underweight” (≤5^th^ percentile), “at risk of being underweight” (5^th^–15^th^ percentile), “normal weight” (15^th^–85^th^ percentile), “at risk of being overweight” (85^th^–95^th^ percentile), and “overweight” (≥95^th^ percentile). The calculation of BMI percentiles for age and sex was based on the standard growth charts developed from the Japanese national survey conducted in 2000 [36].

#### Body Weight Perception

BWP was assessed with the question: “What do you think of your current body weight?” Students could respond by selecting “very underweight,” “slightly underweight,” “about the right weight,” “slightly overweight,” or “very overweight.”

#### Self-induced vomiting for the purpose of dieting

The prevalence of self-induced vomiting for the purpose of dieting was assessed with the question: “Have you ever intentionally vomited (thrown up) after eating in order to lose weight?” Students could select “yes” or “no.”

#### The 12-item General Health Questionnaire

GHQ-12 is widely used as a self-report psychiatric screening test for depression and anxiety [37]. The binary scoring method applied to four-point scale (0011) is used for each of the 12 questions. A total score, which is the sum of all the “1” responses, ranges from 0 (best possible) to 12 (worst possible). GHQ-12 was originally developed for adult populations and was later used and validated for younger populations [38], [39]. The validity and reliability of the Japanese version of GHQ-12 have been confirmed [40]. Adolescents with a total GHQ-12 score ≥4 were considered to have poor mental health [38].

#### Other variables

Previous studies indicate that violence and frequent dieting in adolescents may be influenced by other confounding factors such as victimization [7], [8], [26] and substance use [6], [9]–[11], [26]. In our questionnaire, students were asked about their experiences of being bullied (within the past year), violence from adults at home (within the past month), tobacco use (within the past month), alcohol use (within the past month), and recreational drug use (ever). The questions regarding victimization (“being bullied” and “violence from adults in the home”) and substance use (tobacco and alcohol use and recreational drug use) were answered with either “yes” or “no.” Other variables also included demographic characteristics such as “only child” and “family type.”

### Statistical analysis

Logistic regression analyses were used to estimate associations between violent behavior and the number of diets. Dependent variables were interpersonal violence perpetration and violence toward objects. Multiple-variable analyses were conducted, adjusting for age, the BMI and BWP categories, the GHQ-12 total score, victimization by “being bullied” and “violence from adults in the home,” tobacco and alcohol use, and recreational drug use. In the logistical regression analyses, odds ratios (OR) and 95% confidence intervals (95% CI) were calculated, setting the 15^th^–85^th^ percentiles of the BMI category and the “about the right weight” response of the BWP category as references. For all statistical tests, a two-tailed *p*-value of <0.05 was considered statistically significant. For background information, the rates of the BMI and BWP categories were calculated by interpersonal violence perpetration and violence toward objects, respectively. All statistical analyses, including the descriptive statistics, were conducted using PASW Statistics version 20 for Windows (IBM Software Japan, Tokyo, Japan).

## Results

Of 19,436 students from 45 of the 138 junior high schools and 28 of the 36 high schools, 798 (4.1%) were absent on the survey days and 388 (2.0%) did not agree to participate in the study. Of these 18,250 students (93.9%) that returned the questionnaire, 18,104 (93.1%) gave available responses. Their mean age was 15.3 (SD = 1.7) and ranged 12–18 years. Two investigators on the study team (NS and NW) classified ten cases with unlikely height and weight (e.g., 15.5 cm and 4.5 kg) as having missing values. Consequently, 9,112 females (50.3%) of the 18,104 students were analyzed.

**Figure 1:**
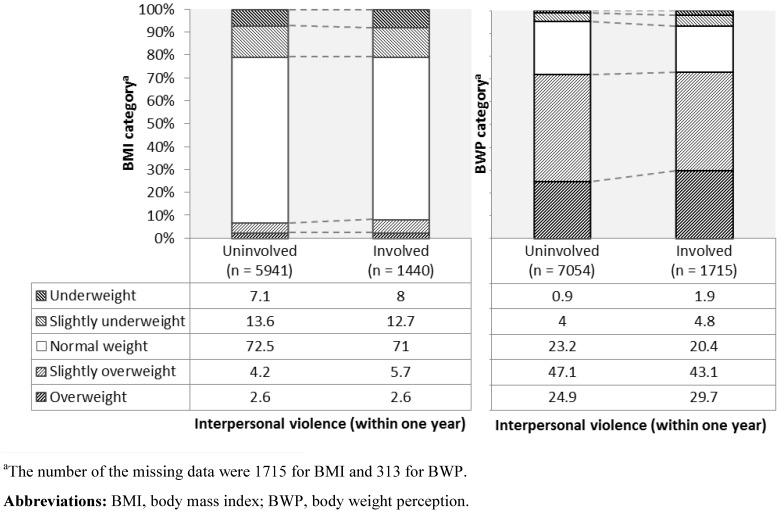
Percentages for the BMI and BWP categories in adolescent females by interpersonal violence perpetration.

**Figure 2:**
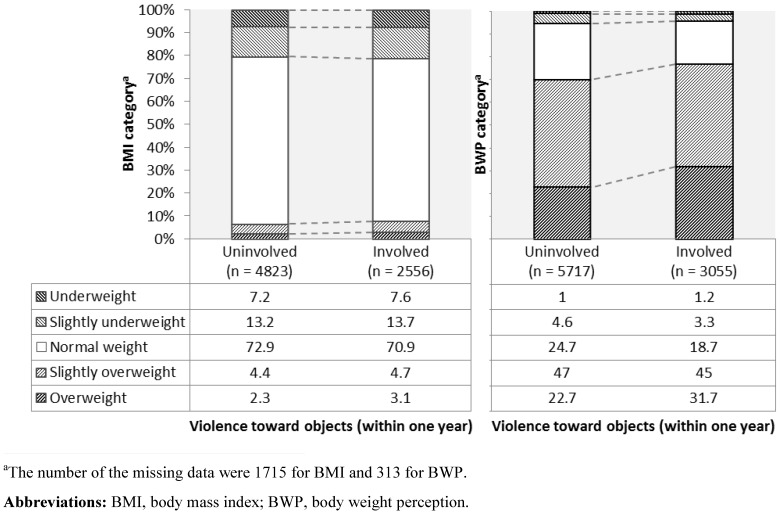
Percentages for the BMI and BWP categories in adolescent females by violence toward objects.

Table 1 presents percentages for demographics and psychological and behavioral problems by violent behavior. Next, Figures 1 and 2 show percentages for the BMI and BWP categories by interpersonal violence perpetration and violence toward objects, respectively. The incidence rates of interpersonal violence perpetration and violence toward objects were 19.6% (1,776/9,065) and 34.9% (3,167/9,068), respectively. Meanwhile, the distribution of the number of diets was as follows: never  = 60.2% (n = 5,481), 1 time  = 7.4% (n = 670), 2 times  = 10.9% (n = 995), 3 times  = 8.3% (n = 759), 4 times  = 1.5% (n = 134), 5 times  = 3.9% (n = 354), 6 times  = 0.5% (n = 48), 7 times  = 0.2% (n = 20), 8 times  = 0.1% (n = 12), 10 times  = 1.6% (n = 146), 12 times  = 0.0% (n = 2), more than 13 times  = 0.8% (n = 74), missing values  = 4.6% (n = 417).

**Table 1:** Percentages for demographics and psychological and behavioral problems in adolescent females by violence.

	Uninvolved^a^		Interpersonal violence perpetration^a^		Violence toward objects^a^	
	n	(%)	n	(%)	n	(%)
Total (aged 12–18 years)	5218	(100)	1776	(100)	3167	(100)
Junior high school (aged 12–15 years)	2179	(41.8)	1065	(60.0)	1552	(49.0)
High school (aged 15–18 years)	3039	(58.2)	711	(40.0)	1615	(51.0)
Only child	396	(7.6)	109	(6.1)	245	(7.7)
Living with both parents	4030	(77.2)	1335	(75.2)	2343	(74.0)
Living with one parent	978	(18.7)	375	(21.1)	689	(21.8)
Living apart from parents	210	(4.0)	66	(3.7)	135	(4.3)
Experience of dieting (ever)	1601	(32.0)	703	(41.6)	1375	(45.7)
Self-induced vomiting for the purpose of dieting (ever)	147	(2.8)	132	(7.5)	273	(8.7)
GHQ-12 total score (≥4)	2272	(43.5)	1213	(68.3)	2287	(72.2)
Being bullied (within one year)	214	(4.1)	231	(13.2)	347	(11.1)
Violence from adults in the home (within one month)	81	(1.6)	198	(11.2)	220	(7.0)
Tobacco use (within one month)	48	(0.9)	77	(4.5)	138	(4.5)
Alcohol use (within one month)	500	(9.7)	372	(21.3)	671	(21.6)
Use of recreational drugs (ever)	12	(0.2)	23	(1.3)	27	(0.9)

a The number of the missing values in each variable was 64 for uninvolved, 47 for interpersonal violence perpetration and 44 for violence toward objects.

**Abbreviations**: GHQ-12, 12-item General Health Questionnaire.

The increased number of diets was associated with the increased prevalence of both interpersonal violence perpetration (OR = 1.18, 95% CI 1.08–1.29, *p*<0.001) and violence toward objects (OR = 1.34, 95% CI 1.24–1.45, *p*<0.001), after adjusting for age, BMI, BWP, the GHQ-12 total score, victimization, and substance use (Table 2). In terms of the categories of BMI and BWP, the “overweight” BWP was associated with violence toward objects (OR = 1.29, 95% CI 1.07–1.54, *p*<0.05). On the other hand, the “Underweight” and “Slightly underweight” BMI were related to violence toward objects [(OR = 1.28, 95% CI 1.01–1.62, *p*<0.05) and (OR = 1.27, 95% CI 1.07–1.51, *p*<0.05), respectively]. The “Underweight” BWP was related to interpersonal violence perpetration (OR = 2.30, 95% CI 1.38–3.84, *p*<0.05). Except for recreational drug use, all other factors were independently associated with both interpersonal violence perpetration and violence toward objects. Recreational drug use was significantly related to interpersonal violence perpetration, but not to violence toward objects.

**Table 2:** Associations between violent behavior and age, the number of diets, BMI, and BWP in adolescent females.^a^

	Interpersonal violence perpetration		Violence toward objects	
	Crude OR (95%CI)	Adjusted OR b (95% CI)	Crude OR (95%CI)	Adjusted OR b (95% CI)
Age	0.80 (0.77–0.82)**	0.76 (0.73–0.79)**	0.93 (0.91–0.96)**	0.85 (0.82–0.88)**
The number of diets (ever)	1.24 (1.16–1.32)**	1.18 (1.08–1.29)**	1.52 (1.43–1.61)**	1.34 (1.24–1.45)**
BMI category				
Underweight	1.13 (0.91–1.40)	1.02 (0.77–1.34)	1.07 (0.89–1.28)	1.28 (1.01–1.62)*
Slightly underweight	0.93 (0.78–1.10)	0.94 (0.76–1.15)	1.04 (0.90–1.20)	1.27 (1.07–1.51)*
Normal weight	1.00	1.00	1.00	1.00
Slightly overweight	1.38 (1.06–1.78)*	1.20 (0.90–1.62)	1.07 (0.85–1.35)	0.92 (0.70–1.19)
Overweight	0.98 (0.69–1.42)	0.93 (0.62–1.40)	1.36 (1.02–1.82)*	1.13 (0.80–1.58)
BWP category				
Underweight	2.14 (1.40–3.27)**	2.30 (1.38–3.84)*	1.21 (0.80–1.82)	1.11 (0.68–1.83)
Slightly underweight	1.24 (0.96–1.59)	1.26 (0.92–1.72)	0.72 (0.57–0.90)*	0.78 (0.58–1.04)
About right	1.00	1.00	1.00	1.00
Slightly overweight	0.85 (0.77–0.95)*	1.01 (0.85–1.19)	0.92 (0.85–1.01)	1.13 (0.98–1.31)
Overweight	1.28 (1.14–1.43)**	1.04 (0.84–1.29)	1.58 (1.43–1.74)**	1.29 (1.07–1.54)*

a The sample size was 6908 for interpersonal violence perpetration and 6903 for violence toward objects depending on the missing date that have been excluded from the statistical analyses.

b ORs adjusted for GHQ-12 total score, being bullied and violence from adults, tobacco and alcohol use, and the use of recreational drugs.

*p<0.05;

**p<0.001.

**Abbreviations**: BMI, body mass index; BWP, body weight perception; OR, odds ratio; CI, confidence interval.

## Discussion

To the best of our knowledge, this is the first study to examine whether repeated dieting is associated with violent behavior. We found a relationship between an increased number of diets and the incidence of interpersonal violence perpetration and violence toward objects in a local representative sample of female adolescents (n = 9,112). The incidence rate of interpersonal violence perpetration (19.6%) in this study was comparable to the rate of the national youth risk behavior survey conducted in the United States (24.4%) [3].

Maladaptive weight-loss behavior accompanied by frequent dieting could have an impact on the occurrence of violence. Maladaptive behavior classified as moderate includes skipping meals, eating a very little amount of food, and using food substitutes. Female dieters with moderate behavior are at risk of developing severe behavior such as self-induced vomiting and the use of laxatives, diuretics, and diet pills [41]. Chronic dieters tend to show severe behavior [42]. The maladaptive weight-loss behavior increase the risk of poor intake of grains, calcium, iron, vitamin B-6, folate, and zinc among female adolescents [43]. Among these nutritional insufficiencies, iron deficiency is the most common and can lead to irritability in young people [44], [45]. Accordingly, a lack of basic nutrients resulting from chronic dieting can potentially cause violent behavior.

Apart from weight-control behavior, the perception of being overweight was associated with violent behavior. Dieting was related to not only an overweight condition but also body-image dissatisfaction [30]. Among female adolescents, the perception of being overweight was more common than having a body weight that was considered overweight [14]. This gap can cause dissatisfaction in their body shape and as a result, they begin dieting [46]. The “overweight” BWP is considered to provoke frustration that may be related to violence toward objects. On the other hand, the both actually being underweight and perception of being underweight are associated with violent behavior. These associations suggests that the physical and mental state of low body-weight can relate to irritability [26], emotional dysregulation, poor impulse control [27], [28] independently. However, these speculations are weakened by the fact that a large sample size could find statistically significant results, even with small differences. In contrast, the cumulative number of diets showed a consistent and strong relationship with violent behavior.

The present study has several limitations. First, our study design was cross-sectional, which made it impossible to prove a causal connection. It means that violent behavior occurred before the onset of a weight-loss diet among some participants. That is, our results indicate that violent behavior might predict underweight BMI, extreme BWP, and repeated dieting. Second, although the GHQ-12 is a valid measure for the severity of depression and anxiety and the total score was used in logistic regression analysis, the confounding effect of common adolescent psychiatric disorders (i.e. ADHD and conduct disorder [9], [12], [20], [21]) was not taken into account. It is also possible that observed associations are attributed to childhood abuse as a confounding factor [17], [18]. This is because victimization, particularly in childhood, is considered as a cause that predisposes female adolescents to both violence perpetration [7], [10] and disordered eating [18], [47]. In spite of our attempt to control for victimization, this control was not sufficient due to the short investigation period. In addition, we did not handle some violence-related social factors (i.e. low academic performance [6], [7] and parent–child and peer relationship [8]–[10]) as potential confounding factors. Consequently, an over- or under-estimation of the association is possible. Third, because this survey was performed in schools, responses from absent students were unavailable. Young people who are engaged in violent behavior and/or repeated dieting may be more likely to be absent from school than those who are not. Finally, the term “weight-loss diet” was not precisely defined, and thus, it included various methods and periods of weight-loss behaviors that female adolescents subjectively interpreted.

In conclusion, the cumulative number of diets is associated with both interpersonal violence perpetration and violence toward objects in female adolescents. In addition, underweight BMI and extreme BWP are associated with violent behavior. Therefore, this study suggested that the subjective number of diets may be clinically useful as a screening marker for violent behavior in female adolescents. Further prospective studies are required to investigate the mechanism of repeated dieting that can lead to violent behavior in female adolescents.
